# Quercetin Attenuates Diabetic Peripheral Neuropathy by Correcting Mitochondrial Abnormality via Activation of AMPK/PGC-1α Pathway *in vivo* and *in vitro*

**DOI:** 10.3389/fnins.2021.636172

**Published:** 2021-03-03

**Authors:** Qian Zhang, Wei Song, Bingjia Zhao, Jun Xie, Qing Sun, Xiaohu Shi, Bin Yan, Guoqing Tian, Xiaochun Liang

**Affiliations:** ^1^Department of Traditional Chinese Medicine, Peking Union Medical College Hospital, Peking Union Medical College, Chinese Academy of Medical Sciences, Beijing, China; ^2^Medical Research Center, Peking Union Medical College Hospital, Peking Union Medical College, Chinese Academy of Medical Sciences, Beijing, China

**Keywords:** diabetes, diabetic peripheral neuropathy, quercetin, AMPK, PGC-1 alpha

## Abstract

The AMPK/PGC-1α pathway-mediated mitochondrial dysfunction has been supposed to play a crucial role in pathogenesis of diabetic peripheral neuropathy (DPN). The present study investigated the neuroprotective potential of quercetin, a natural AMPK activator. Streptozotocin (STZ)-induced diabetic rats that developed DPN phenotype were orally administrated with quercetin (30 and 60 mg/kg per day) for 6 weeks. The morphologic changes in the sciatic nerves (SN), the pathological structure of neurons in dorsal root ganglion (DRG), and the expressions of myelin proteins were assessed. The ATP content and the mitochondrial ultrastructure were measured. Furthermore, key proteins in the AMPK/PGC-1α pathway were determined. As a result, quercetin administration at both doses improved the paw withdrawal threshold, nerve conduction velocity, and the pathologic changes in SN and DRG of DPN rats. The expressions of myelin basic protein and myelin protein zero were also increased by quercetin. The oxidative stress, decreased ATP generation, and morphological changes of mitochondria were corrected by quercetin. *In vitro* study found that quercetin treatment significantly decreased the high-glucose-induced generation of reactive oxygen species, as well as attenuated the mitochondrial morphologic injuries and oxidative DNA damages of RSC96 cells. Quercetin treatment promoted the expressions of phosphorylated AMPK, PGC-1α, SIRT1, NRF1, and TFAM under hyperglycemic state *in vivo* and *in vitro*. This study revealed that the neuroprotective effect of quercetin was mainly related to mitochondrial protection by activation of the AMPK/PGC-1α pathway for the first time and proved quercetin as a potential therapeutic agent in the management of diabetic neuropathy.

## Introduction

Diabetic peripheral neuropathy (DPN), a long-term complication of diabetes, affects more than 50% diabetic patients (Feldman et al., 2017). DPN leads to neuropathic pain, abnormal sensation, and loss of life quality in patients. It not only increases the risk factor of lower-limb amputation (Holman et al., 2012; Boulton, 2013; Vas and Edmonds, 2016) but also contributes to diabetes-related and all-cause mortality in people with diabetes (Hsu et al., 2012). The development of DPN is attributed to multiple factors, such as polyol pathway flux, protein kinase C activation, advanced glycation end product increase, oxidative and inflammatory stress, and insulin pathway disorders (Russell et al., 2002; Schmeichel et al., 2003; Román-Pintos et al., 2016; Goncçalves et al., 2017). Nevertheless, few specific treatments for nerve damage are currently available in addition to optimized glycemic control as early as possible (Farmer et al., 2012; Pop-Busui et al., 2017; American Diabetes Association, 2019). A substantial proportion of diabetic patients develop DPN despite intensive glycemic control. Thus, new rational treatment is still in demand to prevent or reverse nerve injury of diabetic patients.

It has been recognized that the peripheral nervous system has high demands for energy to meet the requirements of axonal outgrowth and myelin sheath regeneration (Fernyhough, 2015; Goncçalves et al., 2017). Mitochondrion is the powerhouse of cells and converts oxygen and gluconic metabolites into adenosine triphosphate (ATP), which serves as a direct source of energy for cellular work. Aberrant function of mitochondrion has been proposed to cause nerve degeneration under metabolic stress induced by hyperglycemia (Vincent et al., 2010; Roy Chowdhury et al., 2011; Fernyhough, 2015; Chung et al., 2018). The adenosine 5′-monophosphate (AMP) activated protein kinase (AMPK)/peroxisome proliferator-activated receptor-γ coactivator 1α (PGC-1α) signaling perceives the cellular energy status and modulates the mitochondrial biogenesis, which plays a pivotal role in regulating energy metabolism and mitochondrial function (Choi et al., 2014; Fernyhough, 2015; Liu et al., 2017). The up-regulation of the AMPK/PGC-1α pathway has been proved to be conducive to the preservation of mitochondrial function in the peripheral nervous system under hyperglycemic states (Yerra et al., 2017, 2018; Aghanoori et al., 2019). Herein, the AMPK/PGC-1α pathway shows novel potential as a therapeutic target for DPN.

**FIGURE 1:**
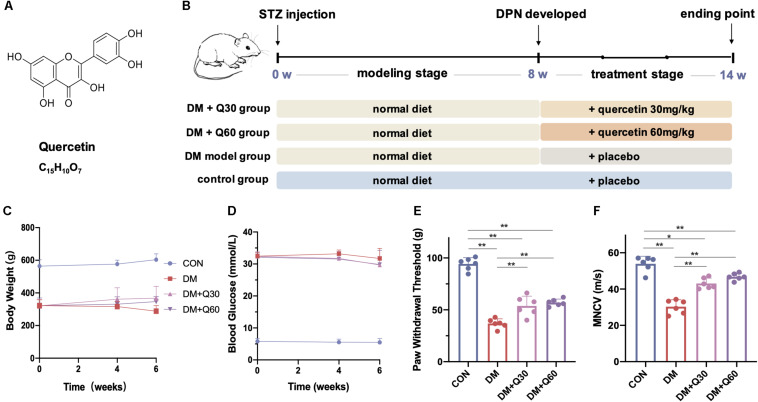
The chemical structure of quercetin **(A)** and the experimental design *in vivo* **(B)**. The body weight **(C)** and blood glucose **(D)** were monitored during the quercetin treatment of 6 weeks, and the effect of quercetin on paw withdrawal threshold **(E)** and motor nerve conduction velocity (MNCV) **(F)** were detected at the end of the 6-week treatment. Data are expressed as mean ± SEM (*N* = 6). *p < 0.05; **p < 0.01. CON, control; DM, diabetes mellitus; Q30, quercetin at 30 mg/kg; Q60, quercetin at 60 mg/kg.

Quercetin is a natural flavonol (Figure 1A), which was widely known for its various bioactivities, such as anti-tumor, anti-oxidation, anti-infection, cardiovascular protection, and so on (Batiha et al., 2020). Previous studies have paid attention to the neuroprotective effect of quercetin, including the promotion of peripheral axon regeneration after sciatic nerve-crush injury (Chen et al., 2017), the inhibition of neuronal apoptosis after cerebral ischemia/reperfusion (Pei et al., 2016), as well as ameliorating neuronal damage induced by high glucose (Shi et al., 2013; Dong et al., 2014; Pei et al., 2016; Zhang et al., 2016). It has been found that quercetin improved the mechanical withdrawal threshold and thermal withdrawal latency of type 2 diabetic rats (Yang et al., 2019). However, the protective effect of quercetin against DPN has not been widely recognized. On the other hand, increasing evidences have revealed that quercetin is a natural AMPK activator. For example, quercetin was able to activate AMPK in rat skeletal muscle cells (L6 myotubes) by increasing the cellular AMP/ATP ratio (Dhanya et al., 2017), as well as to induce AMPK activation in vascular smooth muscle cells via promoting the phosphorylation of the upstream LKB1 (Kim et al., 2018). However, whether quercetin can target the AMPK/PGC-1α pathway to improve DPN remains unknown.

Although previous studies have discovered the neuroprotective potential of quercetin on DPN (Anjaneyulu and Chopra, 2003; Yang et al., 2019), we further explored the underlying molecular mechanism, in which the mitochondrial function and AMPK/PGC-1 α axis were explicitly estimated. In this study, we investigated the effect of quercetin on peripheral neuropathy using streptozotocin (STZ)-induced diabetic rats and the rat Schwann cells line (RSC96) exposed to high glucose. Furthermore, the modulation of mitochondrial function via AMPK/PGC-1α signaling by quercetin was assessed. The present study lays foundation for the neuroprotective mechanism of quercetin and provide a promising therapeutic strategy for DPN.

## Materials and Methods

### Animal and Treatment

The animal experiment has been approved by the Institutional Animal Care and Use Committee of the Peking Union Medical College Hospital (No. XHDW-2019-011) and the protocol was performed in compliance with the Guidelines of the Care and Use of Laboratory Animals issued by the Chinese Council on Animal Research. Male Sprague–Dawley rats (200–220 g) purchased from Vital River Laboratory Animal Technology (Beijing, China) were housed and cared in a pathogen-free environment of the Experimental Animal Center of the Peking Union Medical College Hospital (Beijing, China), with ad libitum access to food and water on a 12-h light–dark cycle schedule. Diabetes was induced by a single intraperitoneal injection of 60 mg/kg STZ (Sigma, St. Louis, United States) after an overnight fast (Al-Awar et al., 2016; Yan et al., 2018). Blood glucose was measured from the tail tip using the Accu-Chek glucose meter (Roche, Fermoy, Ireland). Diabetic rats were confirmed with blood glucose ≥ 16.7 mmol/L after 72 h injection (Yang et al., 2015).

After modeling, the diabetic rats were randomly assigned into three groups (*N* = 6 per group): the DM model group, quercetin low-dosage (30 mg/kg) treated group (DM + Q30), and quercetin high-dosage (60 mg/kg) treated group (DM + Q60). Quercetin (Sigma, St. Louis, United States) was intragastrically administrated at 30 mg/kg per day for rats in the DM + Q30 group and 60 mg/kg for rats in the DM + Q60 group after 8 weeks of STZ injection, and lasted for a further 6 weeks. Meanwhile, six age-matched control rats with blood glucose < 7.0 mmol/L in the control group (CON) and rats in the DM model group received the same volume of distilled water.

At the end point of the experiment, all rats were euthanized and sacrificed by intraperitoneal injection with 10% chloral hydrate (3 ml/kg). Blood samples collected from the carotid artery were centrifuged at 3000 r/min for 15 min, and the plasma specimens were stored at −80°C. The left sciatic nerves (SN) were obtained for pathomorphological detection and the right side was frozen in liquid nitrogen and stored at −80°C for biochemical analysis. The right lumbar dorsal root ganglia (DRG) were harvested and fixed in 10% formalin for Nissl staining by toluidine blue and immunohistochemical assay.

### Mechanical Hyperalgesia

After 6 weeks of treatment, mechanical allodynia was assessed by paw withdrawal threshold using the Von Frey Pain Measurement Instrument (IITC Life Science Inc., United States) as described previously (Zhang et al., 2020). The hind paw was stimulated vertically by a probe and the force at which paw withdrawal was recorded in grams. Each rat was measured three times on bilateral hind paws with a 5-min interval and the paw withdrawal threshold was considered as the average measurements.

### Motor Nerve Conduction Velocity (MNCV)

The determination of MNCV of SN was performed according to the previously described method (Ota et al., 2013). The rats under anesthesia were kept with body temperature at 37.5°C–38.5°C. With a pair of monopolar needle electrodes (1.0–1.5 mA, 2.0 mV/D), the active electrode S1 was situated in the right sciatic notch of the sciatic nerve as the proximal stimulation point, and the active electrode S2 was put in the ankle region of the right tibial nerve as the distal stimulation point. Responses were recorded from the plantar muscles and the latencies of M-wave (t1 and t2) were obtained using an EMG/EP system (NCC Medical Co. Shanghai, China). The distance (d) between S1 and S2 was measured with a caliper. The MNCV was determined using the equation: MNCV (m/s) = d/(t1 – t2).

### Transmission Electron Microscopy (TEM)

The left SN (2–3 mm) was fixed with 2.5% glutaraldehyde at 4°C for 24 h. The processing for TEM followed the routine methods. The segment of SN was rinsed in 0.1 M PBS, osmicated, fixed in 1% osmium tetroxide, dehydrated, and then embedded in epoxy resin. Seventy-nanometer-thick sections were cut with an ultramicrotome. After counterstaining with uranyl acetate and lead citrate, TEM-1400 plus (Tokyo, Japan) was used to observe pathological changes. As described previously (Shin et al., 2017; Wu et al., 2018), morphological parameters including the percentage of abnormal myelinated fibers, G ratio (axon diameter/myelinated fiber diameter), axon diameter and myelinated fiber diameter were assessed using the ImageJ software (version 1.47, United States). More than 150 axons (15–20 images) from three rats in each group were used for measuring these parameters. The quantified density of mitochondria was determined as the number of mitochondria per 1 μm^2^ as previously described (Choi et al., 2014). Mitochondria that were defined as vacuolar structures with a double membrane and clear cristae structure were calculated using TEM at 32,000 times magnification. Mitochondria from 10 random sections were measured from the SN of each rat and were averaged across the individual and all sections for each animal.

### Oxidative Stress and Antioxidant Capacity Analysis

The lipid peroxidation indicator malondialdehyde (MDA) in plasma and SN homogenates were measured by quantifying the thiobarbituric acid reactive substance using the MDA assay kit (Nanjing Jiancheng, Nanjing, China). The absorbance was set at 532 nm. The reduced glutathione (GSH) and the total antioxidant capacity (TAOC) were also tested, as both of them indicate the activity of antioxidant defense system. According to Ellman’s method (Moron et al., 1979), GSH in plasma and SN homogenates were measured using the GSH assay kit (Nanjing Jiancheng, Nanjing, China). The absorbance was set at 420 nm. TAOC was detected by the ferric-reducing antioxidant power reaction method according to the protocol of the TAOC assay kit (Nanjing Jiancheng, Nanjing, China). The absorbance was set at 520 nm.

### Immunohistochemical Assay of Myelin Protein

After dehydration with graded alcohol, DRG samples were embedded in paraffin and cut into 5-μm sections. The sections were placed in EDTA antigen repair solution (pH 9.0) for 10 min to repair hot antigens, put into 3% hydrogen peroxide solution to block endogenous peroxidase, and then incubated in blocking solution containing 3% BSA (Solarbio, China) for 30 min. The sections were incubated with the primary antibody rabbit polyclonal to rat myelin protein zero (MPZ, 1:200, Abcam) and mouse anti-rat myelin basic protein (MBP, 1:200, R&D Systems) at 4°C overnight and then incubated with the HRP-labeled secondary antibody (1:100). After color development by DAB, the nuclei were retained by Harris hematoxylin and finally dehydrated and sealed the slices. Images were captured by the Nikon DS-U3 image acquisition system (Nikon, Japan). Image-Pro Plus (version 7.0, United States) was used for image analysis. The average optical density (AOD) was used to semi-quantify the protein level.

### Western Blot (WB) Analysis

The sciatic nerves were homogenized with radioimmunoprecipitation assay (RIPA) lysis buffer (Sigma, St. Louis, United States) on ice, and total protein concentration in each sample was measured by the BCA Protein Assay method (Thermo Scientific, Waltham, MA, United States). Each sample containing the same amount of protein was loaded and separated in 10% SDS-PAGE and transferred on PVDF (Millipore, Bedford, United States) membranes. After blocking with 5% fat-free milk at room temperature for 1 h, the membranes were washed with Tris-buffered saline with 0.1% Tween (TBST), incubated with primary antibody: P-AMPK (Thr172) (1:1000), AMPKα (1:1000), Sirt1 (1:1000), NRF1 (1:1000) (Cell Signaling Technology, MA, United States), PGC-1α (1:1000), and TFAM (1:2000) (Abcam, MA, United States) at 4°C overnight, followed by washing and the incubation of the HRP-conjugated secondary antibody for 1 h. The membranes were further rinsed. The immunoreactive areas were visualized using enhanced chemiluminescence (ECL kit, MA, United States). Relative optical densities were quantified by ImageJ software (version 1.47, United States).

### *In vitro* Studies

#### Cell Culture and Treatment

The rat Schwann cell line (RSC96) was purchased from National Infrastructure of Cell Line Resource (Beijing, China). The cells were cultured in Dulbecco’s Modified Eagle’s Medium culture medium (Gibco, NY, United States) containing 10% fetal bovine serum with 5% CO_2_ and 95% air at 37°C. According to our preliminary studies, high glucose injury was stimulated by addition glucose to reach the final glucose concentration of 100 mM, while the normal glucose condition contained 25 mM glucose (Supplementary Figure 1). Quercetin was dissolved in DMSO at optimized concentration and stored at −20°C in the dark. The cells were divided into five groups: the normal-glucose group (NG), high-glucose group (HG), quercetin-treated high-glucose group (HG + Q), A769662 (AMPK activator)-added high-glucose group (HG + A), and quercetin and dorsomorphin (Compound C, an AMPK inhibitor) co-treated high-glucose group (HG + Q + CC). NG and HG groups were treated with DMSO for 48 h after incubation under normal-glucose and high-glucose conditions, respectively. The HG + Q group was treated with quercetin for 48 h after incubating under high-glucose conditions for 48 h, while the HG + A group was treated with A769662 and the HG + Q + CC group was co-treated with quercetin and Compound C for 48 h after incubating under high-glucose conditions for 48 h.

#### Measurement of Intracellular Reactive Oxygen Species

Intracellular reactive oxygen species (ROS) were determined with the ROS Assay Kit (Nanjing Jiancheng, Nanjing, China) according to the manufacturer’s protocol. After incubation for 48 h, cells were treated with different concentrations of quercetin (5, 10, and 20 μM) for another 48 h. The cells were rinsed with PBS and then incubated with oxidation-sensitive fluoroprobe 2′,7′-dichlorofluorescin diacetates (DCFH-DA) at a concentration of 10 μM for 30 min at 37°C. The fluorescence intensity was measured at 500 nm excitation and 525 nm emission.

#### Cell Proliferation Activity

The cell proliferation activity was measured by Cell Counting Kit-8 (CCK8) assay (Nanjing Jiancheng, Nanjing, China). Ten microliters of the CCK8 solution was transferred to each well and incubated for 1 h at 37°C. The cell viability was measured at 450 nm. The mean optical density values were applied to determine the cell viability using the following formula: percentage of cell viability = (A treatment group – A blank group)/(A control group – A blank group) × 100%.

#### TUNEL Assay

RSC96 apoptosis was detected by TUNEL assay. RSC96 were fixed with 4% paraformaldehyde and incubated with 0.1% Triton X-100 in 0.1% sodium citrate for 2 min on ice. After centrifuging and washing, the cells were incubated with fluorescein isothiocyanate (FITC)-labeled nucleotides and terminal deoxy nucleotidyl transferase (TdT) enzyme (Roche, Germany) at 37°C for 60 min in a humidified chamber. The cells were at last mounted with DAPI mountant before they are observed under a fluorescence microscope (apoptotic cells show green staining and total nuclei was blue labeled by DAPI). Images from different fields were captured by a fluorescent microscope (Nikon Eclipse Ti-SR, Japan) with a Nikon DS-U3 digital system. The average of TUNEL-positive cells was expressed as percentage of total number of cells.

#### Assessment of Mitochondrial Structure by TEM

After exposure to the above different conditions, the cells were dissociated by trypsin. The cellular suspension was centrifuged at 1000 rpm for 5 min. The samples were fixed with 2.5% glutaraldehyde, washed with PBS, and post-fixed with 1% osmium tetroxide for 1 h. Passing through the procession of dehydration in graded alcohol and acetone, the samples were embedded in epoxy resin and sliced in 70-nm sections. The sections were placed on the TEM grids and stained. The ultrastructure of the mitochondria was observed and imaged by TEM-HT7700 (HITACHI, Japan) at 11,000 times magnification. The density of mitochondria was determined as the number of mitochondria per 1 μm^2^. Mitochondria from 10 random fields were measured from each section.

#### WB Analysis

After treatment, the cells were collected, homogenized in RIPA lysis buffer (Sigma, St. Louis, United States), and then centrifugated at 12,000 rpm for 30 min at 4°C. Then, the expression of P-AMPK (Thr172), AMPKα, Sirt1, NRF1, PGC-1α, and TFAM was determined by the method as described in Section “Western Blot (WB) Analysis.”

### Statistical Analysis

Statistical analysis was performed using GraphPad Prism (version 8.2, La Jolla, United States). Data were expressed as mean ± SEM. Differences between groups were compared by one-way analysis of variance (ANOVA) with *post hoc* test of LSD. Significance was set at *p* < 0.05.

## Results

### Animal Experiments

Previous studies have proved that diabetic rats displayed neuropathy phenotype after 8 weeks of STZ injection (Yerra et al., 2018). Thus, 8-week STZ-induced diabetic rats were considered as DPN model rats in this study. A 6-week administration of quercetin at two different dosages was then carried out (Figure 1B).

#### Effect of Quercetin on Body Weight, Blood Glucose, Paw Withdrawal Threshold, and MNCV

Oral administration of quercetin (30 and 60 mg/kg *p.o.*) for 6 weeks showed no significant impact on body weight (*p* > 0.05) or blood glucose (*p* > 0.05) on diabetic rats (Figures 1C,D). The paw withdrawal threshold was significantly decreased in diabetic model rats when compared with age-matched healthy rats at 14th week (*p* < 0.01), while administration of quercetin for 6 weeks significantly increased the paw withdrawal threshold (*p* < 0.01, compared to diabetic model rats) (Figure 1E). There was no significant difference on paw withdrawal threshold between the two quercetin-treated groups at different dosages (*p* > 0.05, Figure 1E). When compared with the healthy rats, the MNCV of untreated diabetic rats was significantly decreased at 14 weeks post STZ injection (53.91 ± 2.09 *vs*. 30.29 ± 1.91 m/s) (*p* < 0.01, Figure 1F). Oral administration of quercetin (30 and 60 mg/kg, *p.o.*) for 6 weeks significantly improved the MNCV (43.07 ± 1.48 and 46.78 ± 0.96 m/s at 30 and 60 mg/kg, respectively, *p* < 0.01, compared to diabetic model rats). The difference of MNCV between the two quercetin-treated groups was not significant (*p* > 0.05, Figure 1F).

#### Quercetin Ameliorates the Pathomorphological Characteristics of SN From Diabetic Rats

**FIGURE 2:**
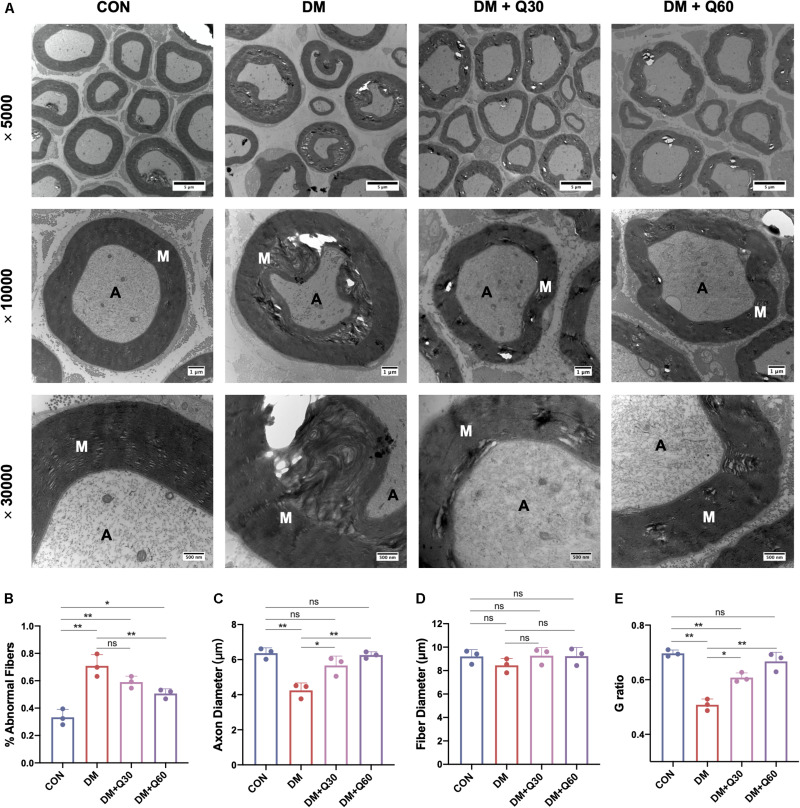
Representative images of transmission electron microscopy of the sciatic nerves **(A)** and the morphological analyses of % abnormal fibers **(B)**, axon diameter **(C)**, fiber diameter **(D)**, and G ratio **(E)**. The control group showed regular myelin sheath with clear lamellar structure while the DM group exhibited demyelination changes (vacuolar-like defects and separation of lamellar structure) and axonal atrophy. In quercetin-treated groups (DM + Q30 and DM + Q60), these demyelination changes and axon atrophy were markedly improved. Panel shows cross sections with 5000×, 10,000×, and 30,000× amplification (scale bar = 5, 1 μm, and 500 nm, respectively). Data are expressed as mean ± SEM (*N* = 3). *p < 0.05; **p < 0.01; ns, no significance with *p* > 0.05. A, axon; M, myelin. CON, control; DM, diabetes mellitus; Q30, quercetin at 30 mg/kg; Q60, quercetin at 60 mg/kg.

As shown in Figure 2A, ultrastructural changes of sciatic nerves were observed using TEM. The axons of myelinated nerve fibers were surrounded by myelin sheath with a clear lamellar structure in normal rats. In contrast, visible signs of demyelination and degeneration of axonal fibers were evident in diabetic rats. The DM model group exhibited obvious vacuolar-like defects and separation of lamellar structure in the myelin sheath, and axonal shrinkage. Quercetin treatment at 30 and 60 mg/kg ameliorated demyelination in myelin sheath and profoundly corrected the axon atrophy. Furthermore, according to morphometric analyses (Figures 2B–E), there was a significant increase in the percentage of abnormal fibers (*p* < 0.01), and decrease of the axon diameter (*p* < 0.01) and G ratio (*p* < 0.01) in SN from rats in the DM model group, as compared to control. The percentage of abnormal fibers in the DM + Q60 group decreased in comparison to the DM group (*p* < 0.01). Axon diameter and G ratio (axon diameter/fiber diameter) were all significantly increased in the DM + Q60 group (*p* < 0.01 for both), when compared with the DM group. The same effect was observed with respect to the DM + Q30 group (*p* < 0.05 for both). There were no differences in fiber diameter between CON, DM, and DM + Q30/Q60 groups.

#### Quercetin Improved Pathological Injury of DRG Neurons

**FIGURE 3:**
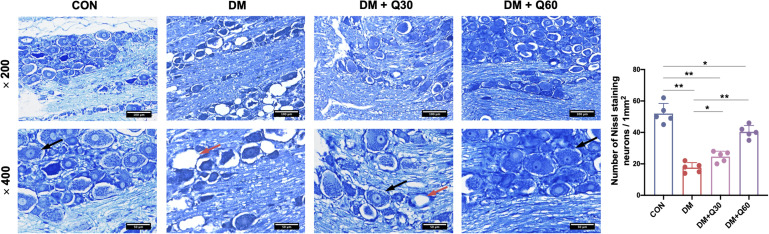
Nissl staining of the dorsal root ganglion (DRG) neurons and quantitative analysis of Nissl-stained neurons. Normal DRG neurons (black arrow) showed clear structure with numerous Nissl bodies in the cytoplasm and nucleoli in the nucleus. DRG from diabetic rats exhibited loss of neurons with vacuolar-like structure (red arrow). The neuron damages were improved in quercetin-treated groups (DM + Q30 and DM + Q60). Panel shows longitudinal sections with 200× and 400× amplification (scale bar = 100, 50 μm). Data are expressed as mean ± SEM (*N* = 5). *p < 0.05; **p < 0.01. CON, control; DM, diabetes mellitus; Q30, quercetin at 30 mg/kg; Q60, quercetin at 60 mg/kg.

According to the Nissl staining of DRG (Figure 3), the structure of neuron body was clear with numerous blue granules in the cytoplasm in the control group. The neuronal structure was disordered in the DM model group, in which the nuclei and nucleoli were blurred. Blue granules in the neuronal cytoplasm stained deeper and showed shrinkage in the DM model group. The loss of neurons was remarkable and many vacuolar-like structures left in untreated diabetic rats. The average number of Nissl-stained neurons in a 1-mm^2^ area was quantified using a light microscope (200×) from five random fields of each rats. As shown in Figure 3, the number of Nissl-stained neurons was significantly decreased in the DM model group when compared with the control group (*p* < 0.01). When compared with the DM model group, quercetin treatment caused a significant increase in amounts of neurons at both 30 and 60 mg/kg (*p* < 0.05 and 0.01, respectively). The morphological damages and loss of DRG neurons evidently attenuated in quercetin treated groups. Furthermore, quercetin at 60 mg/kg showed a better therapeutic effect on neuropathological restoration of DRG neurons.

#### Quercetin Enhanced the Expressions of Myelin Proteins in SN and DRG

**FIGURE 4:**
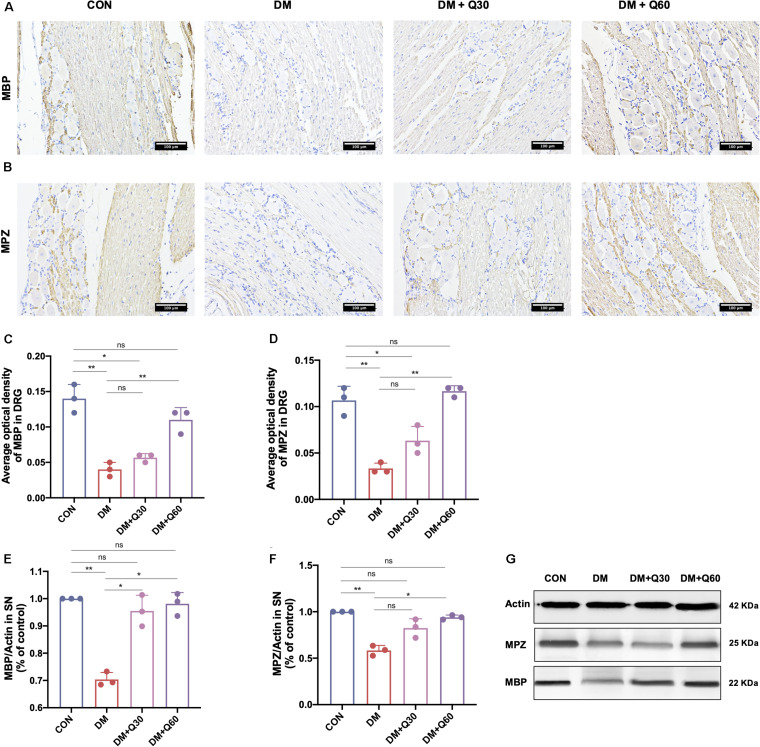
Effect of quercetin on expression of myelin protein myelin basic protein (MBP) and myelin protein zero (MPZ). **(A,B)** Representative immunohistochemical images of MBP and MPZ expression in the dorsal root ganglion (DRG) (×200, scale bar = 100 μm). **(C,D)** Bar graph represents average optical density (AOD) by quantified analyses of MBP and MPZ expression in DRG. **(E,F)** Bar graph shows the quantified Western blot data of MBP and MPZ in the sciatic nerves (SN). **(G)** Representative Western blot images of MBP and MPZ in SN. Data are expressed as mean ± SEM (*N* = 3). *p < 0.05; **p < 0.01; ns, no significance with *p* > 0.05. CON, control; DM, diabetes mellitus; Q30, quercetin at 30 mg/kg; Q60, quercetin at 60 mg/kg.

The immunohistochemical images of DRG in longitudinal sections are shown in Figures 4A,B. Myelin protein zero (MPZ) and myelin basic protein (MBP) were localized in Schwann cells and nerve fibers of DRG. When compared with healthy control rats, MPZ and MBP expressions of DRG were significantly reduced in DM model rats (*p* < 0.01) (Figures 4C,D). According to results of WB (Figures 4E–G), MPZ and MBP levels of SN in the DM model group were significantly decreased (*p* < 0.01) when compared with the control group. Quercetin administration at 60 mg/kg elevated MPZ and MBP levels in both DRG (*p* < 0.01) and SN (*p* < 0.05). Quercetin administration at 30 mg/kg significantly increased the level of MBP in SN (*p* < 0.05), but did not significantly change the levels of MPZ and MBP expression in DRG (*p* < 0.05).

#### Quercetin Restored the Oxidative Stress in SN and Plasma

**FIGURE 5:**
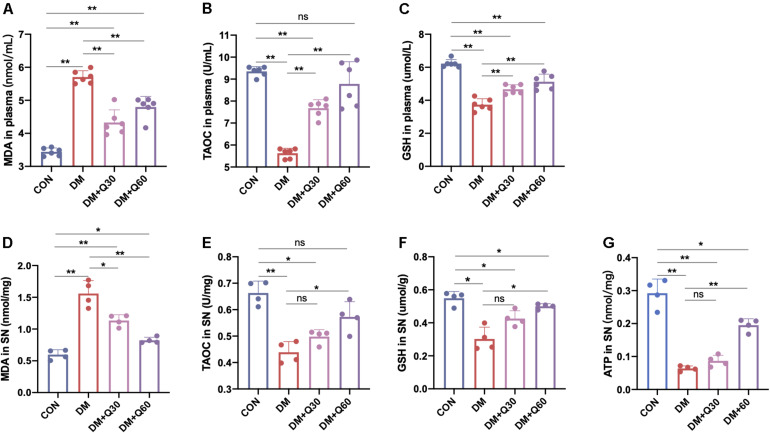
Effects of quercetin on the levels of MDA **(A,D)**, TAOC **(B,E)**, GSH **(C,F)** in plasma and SN, and ATP **(G)** in SN of diabetic rats. Data are expressed as mean ± SEM (**A–C**: *N* = 6; **D–G**: *N* = 4). *p < 0.05; **p < 0.01; ns: no significance with *p* > 0.05. CON, control; DM, diabetes mellitus; Q30, quercetin at 30 mg/kg; Q60, quercetin at 60 mg/kg. MDA, malondialdehyde: TAOC, total antioxidant capacity; GSH, reduced glutathione.

Malondialdehyde level indicates the degree of lipid peroxidation, while the TAOC and GSH reflects the anti-oxidative ability. Hence, MDA, TAOC, and GSH were determined in plasma and SN of the rats (Figure 5). When compared to normal rats, DM model rats showed a significant increase in MDA (*p* < 0.01) and decrease in TAOC (*p* < 0.01) in both plasma and SN. Compared with the DM model group, MDA level in the DM + Q30 group was significantly decreased in plasma (*p* < 0.01) and SN (*p* < 0.05), as well as that in the DM + Q60 group was significantly decreased in plasma (*p* < 0.01) and SN (*p* < 0.01). Compared with the DM model group, TAOC level in plasma of rats in the DM + Q30 group was significantly higher (*p* < 0.01) and that in the DM + Q60 group also elevated in plasma (*p* < 0.01) and SN (*p* < 0.05). There was no statistical significance in TAOC levels in both SN and plasma between the control group and the DM + Q60 group (*p* > 0.05). As for GSH, both the plasma and the SN levels in the DM model group was lower than that in the control group (*p* < 0.01 and 0.05, respectively), which was increased in the DM + Q60 group (*p* < 0.01 and 0.05, respectively). GSH level of plasma in the DM + Q30 group was also higher than that in the DM model group, but there was no statistical significance in GSH levels of SN between the DM + Q30 and DM model group (*p* > 0.05). GSH levels in both plasma and SN were significantly decreased in the DM + Q30 group when compared to the control group (*p* < 0.01 and 0.05, respectively), as well as the GSH levels that were decreased in the DM + Q60 group (*p* < 0.01 and 0.05, respectively).

#### Quercetin Corrected the Mitochondrial Morphological Changes and Decreasing ATP Generation in SN of Diabetic Rats

**FIGURE 6:**
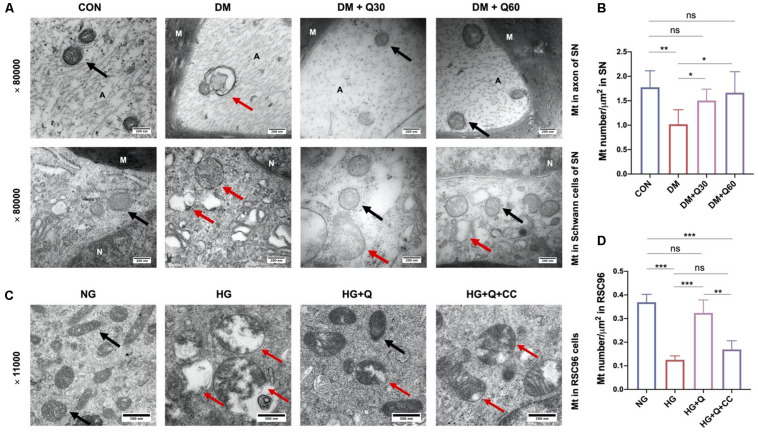
Representative transmission electron microscopy images of mitochondria in the sciatic nerves of rats in CON, DM, DM + Q30, and DM + Q60 groups *in vivo* **(A)**, and mitochondria in rats Schwann cells line (RSC96) in NG, HG, HG + Q, and HG + Q + CC groups *in vitro* **(C)**. The black arrows show normal mitochondria with a double membrane structure and distinct cristae. The red arrows indicate degenerative mitochondria (swelling and vacuolar-like changes, or empty vacuolar structures with double membranes like terminal degeneration). Mitochondria density in the sciatic nerves of rats in different groups *in vivo* **(B)** and in RSC96 cells *in vitro* **(D)**. Data are expressed as mean ± SEM (*N* = 3). *p < 0.05; **p < 0.01; ***p < 0.001; ns, no significance with *p* > 0.05. A, axon; M, myelin; Mt, mitochondria; SN, sciatic nerves; CON, control; DM, diabetes mellitus; Q30, quercetin at 30 mg/kg; Q60, quercetin at 60 mg/kg; NG, normal glucose; HG, high glucose; Q, quercetin; CC, Compound C.

We observed the mitochondrial morphological changes using TEM and measured the ATP concentration in SN to estimate the ability of mitochondria to produce ATP. TEM showed that the mitochondria in both Schwann cells and axon in the control group had regular double membrane contours with clear cristae (Figure 6A). In contrast, swollen and vacuolar-like mitochondria with absent cristae were observed in the DM model group. However, quercetin administration (30 and 60 mg/kg) alleviated mitochondrial degeneration in diabetic rats such as swelling, vacuolar-like defects, and cristae structure destruction. There was a significant decrease in mitochondria density of the diabetic model compared to the control rats (Figure 6B) (*p* < 0.01). Quercetin treatment significantly increased mitochondria density at both 30 and 60 mg/kg (*p* < 0.05). Furthermore, the ATP level of SN decreased significantly in DM model group when compared with the control group (*p* < 0.01). Quercetin administration at 60 mg/kg significantly increased the ATP level in SN when compared with the diabetic rats (*p* < 0.05), indicating that quercetin also accelerated the ATP generation of SN in diabetic rats (Figure 5G).

#### Quercetin Activated the AMPK/PGC-1α Pathway in SN of Diabetic Rats

**FIGURE 7:**
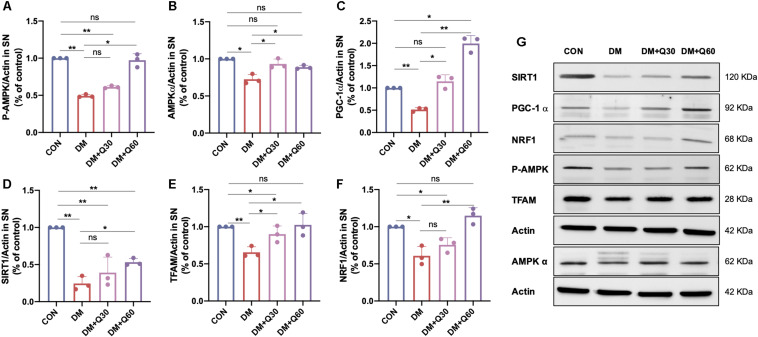
WB examination of the AMPK/PGC-1α pathway-related proteins in the sciatic nerves of rats in CON, DM, DM + Q30, and DM + Q60 groups. Bar graph represents average optical density (AOD) by quantified analyses of blot data of P-AMPK **(A)**, AMPKα **(B)**, PGC-1α **(C)**, SIRT1 **(D)**, TFAM **(E)**, and NRF1 **(F)** expressions in SN, respectively. Data are expressed as mean ± SEM (*N* = 3). *p < 0.05; **p < 0.01; ns, no significance with *p* > 0.05. **(G)** Representative WB images of P-AMPK, AMPKα, PGC-1α, SIRT1, TFAM, and NRF1 expression in SN. SN, sciatic nerves; CON, control; DM, diabetes mellitus; Q30, quercetin at 30 mg/kg; Q60, quercetin at 60 mg/kg.

The protein expression of the AMPK/PGC-1α pathway was further analyzed in SN of rats from different groups by WB. The expressions of SIRT1, phosphorylated AMPK (P-AMPK, Th172), AMPK α, SIRT1, PGC-1α, TFAM, and NRF1 proteins were all decreased in the DM model group when compared with the control group (*p* < 0.05) (Figure 7), indicating that the AMPK/PGC-1α pathway was inhibited in SN of diabetic rats. The decline of all the six proteins were significantly corrected by quercetin treatment at 60 mg/kg (*p* < 0.05). The decreased levels of AMPKα, PGC-1α, and TFAM in diabetic rats were significantly enhanced by quercetin at the dosage of 30 mg/kg (*p* < 0.05), while there is no statistical significance in P-AMPK, SIRT, and NRF1 between the DM + Q30 and DM model group (*p* > 0.05). There was no significant difference in P-AMPK, AMPKα, TFAM, and NRF1 between the control rats and diabetic rats treated with quercetin at 60 mg/kg (*p* > 0.05).

### *In vitro* Studies

Schwann cells of the peripheral nervous system ensheath the axons of myelinated nerve with myelin or embed small axons in their membranes to form a Remak bundle. In the present study, we further tested the impacts of quercetin on rat Schwann cells line (RSC96) that underwent high glucose (HG) irritation *in vitro* for verification. According to the results of proliferation activity and ROS determination, we selected the optimal concentration of quercetin (10 μM) to explore the effect of quercetin on oxidative DNA damage, mitochondrial morphological changes, and the AMPK/PGC-1α pathway protein expressions in HG-exposed RSC96 cells, with comparison to the positive control drug AMPK activator and co-treatment with pathway inhibitor.

#### Quercetin Inhibited ROS Generation and Promoted the Proliferation Activity of HG-Exposed RSC96 Cells

Three concentrations of quercetin (5, 10, and 20 μM) were employed to investigate the effect of quercetin on RSC96 cells exposed to high glucose by proliferation activity and ROS generation. Firstly, high-glucose (100 mM) exposure for 48 h significantly increased ROS level when compared to the normal-glucose (25 mM) exposure to RSC96 cells (*p* < 0.001) (Supplementary Figure 2). Quercetin at three concentrations significantly down-regulated the ROS level in HG-exposed RSC96 cells (*p* < 0.001). There was no significant difference between the HG + Q10 and HG + Q20 group. The cell proliferation activity of HG-exposed RSC96 was significantly suppressed compared with normal-glucose incubated cells (*p* < 0.001). Quercetin at three dosages elevated the cell proliferation activity of RSC96 exposed to high glucose (*p* < 0.05, 0.001, and 0.01, respectively). Moreover, quercetin at higher dosage (10 and 20 μM) significantly up-regulated the proliferation activity of RSC96 cells exposed to high glucose (*p* < 0.01). Among the three dosages, quercetin at a concentration of 10 μM showed the best effect, and this concentration was selected for subsequent studies.

#### Quercetin Inhibited the Oxidative DNA Damage in HG-Exposed RSC96 Cells

**FIGURE 8:**
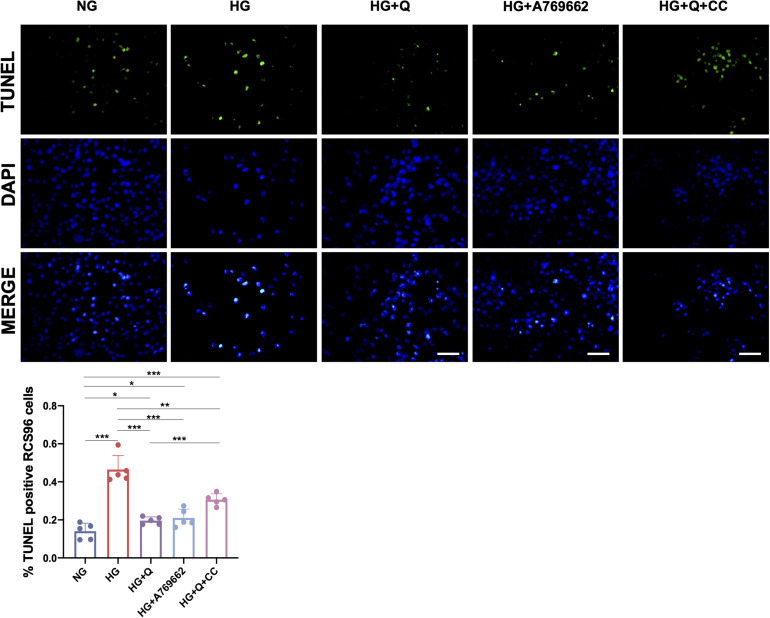
Representative images of TUNEL assay and quantification of TUNEL-positive cells. The green staining area represents the apoptotic cells detected by TUNEL assay, and the blue staining area shows the total nuclei (DAPI) of RCS96 cells (×200 amplification; scale bar = 50 μm). Data are expressed as mean ± SEM (*N* = 5). **p < 0.01; ***p < 0.001. NG, normal glucose; HG, high glucose; Q, quercetin; CC, Compound C.

To investigate the effect of high glucose and quercetin treatment on oxidative DNA damage of RSC96 cells, the TUNEL assay was carried out (Figure 8). The results showed higher percentage of apoptotic cells (TUNEL positive) in the HG group in comparison with the NG group (*p* < 0.001). Both A769662 and quercetin significantly reduced the cell apoptosis under HG conditions (*p* < 0.001), although the apoptotic cells in the HG + Q group was increased when compared with the control NG group (*p* < 0.05). The percentage of the apoptotic cells in the HG + Q + CC group was also lower than the HG group (*p* < 0.01), whereas it was higher than the HG + Q group (*p* < 0.001). The results showed that quercetin inhibited apoptosis of HG-exposed RSC96 cells.

#### Quercetin Alleviated Mitochondrial Ultrastructural Changes of HG-Exposed RSC96 Cells

As shown in Figure 6C, the morphological changes of mitochondria in RSC96 cells were observed by TEM. The mitochondria in the NG group displayed intact double membrane, distinct cristae, and proportional size, while the mitochondria in the HG group exhibited increasing swelling mitochondria, cristae destruction, and vacuolar-like structures with double membranes, which indicated mitochondrial degeneration. However, these degenerative changes of mitochondria were restored in the HG + Q group. Meanwhile, the morphological improvement of mitochondria in the HG + Q + CC group was not as obvious as in the HG + Q group. According to the morphological analysis (Figure 6D), the mitochondria density in the HG group compared to the NG group was significantly decreased (*p* < 0.001). Quercetin treatment caused a significant increase in mitochondria density in the HG + Q group compared to the HG group (*p* < 0.001), while co-treatment with Compound C did not show a similar effect.

#### Quercetin Activated the AMPK/PGC-1α Pathway in HG-Exposed RSC96 Cells

**FIGURE 9:**
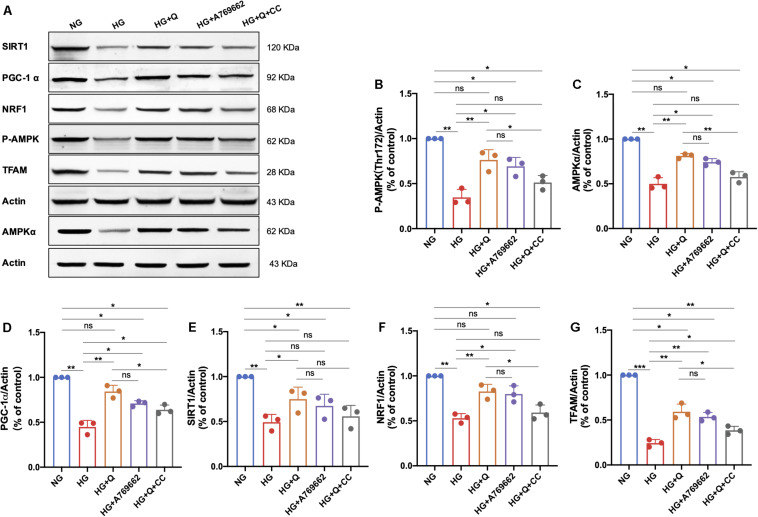
Representative immunoblot images of P-AMPK, AMPKα, SIRT1, PGC-1α, TFAM, and NRF1 protein expression in RSC96 cells of different groups **(A)**. Bar graph represents average optical density (AOD) by quantified analyses of blot data of P-AMPK **(B)**, AMPKα **(C)**, PGC-1α **(D)**, SIRT1 **(E)**, NRF1 **(F)**, and TFAM **(G)** expression in RSC96 cells, respectively. Data are shown in mean ± SEM (*N* = 3). *p < 0.05; **p < 0.01; ***p < 0.001; ns, no significance with *p* > 0.05. NG, normal glucose; HG, high glucose; Q, quercetin; CC, Compound C.

As shown in Figure 9, high-glucose exposure caused a significant decrease in expressions of P-AMPK, AMPKα, PGC-1α, SIRT1, NRF1, and TFAM in RCS96 cells when compared to the NG group (*p* < 0.01). Quercetin administration (10 μM) for 48 h significantly up-regulated the expressions of AMPKα (*p* < 0.01), P-AMPK (*p* < 0.01), PGC-1α (*p* < 0.01), SIRT1 (*p* < 0.05), NRF1 (*p* < 0.01), and TFAM (*p* < 0.01) in HG-exposed RSC96 cells, whereas the AMPK activator A769662 showed coincident effect on these indicators (*p* < 0.05) except SIRT1 (*p* > 0.05). There was no significant difference in expressions of all these six proteins between the HG + A769662 and HG + Q group (*p* > 0.05). Additionally, there was no statistical difference in protein levels of AMPKα, p-AMPK, SIRT1, and NRF1 between the HG + Q + CC group and the HG group (*p* > 0.05). However, the addition of the AMPK inhibitor (Compound C) still increased the protein level of PGC-1α and TFAM significantly (*p* < 0.05). Meanwhile, the protein level of P-AMPK (*p* < 0.05), AMPKα (*p* < 0.01), PGC-1α (*p* < 0.05), NRF1 (*p* < 0.05), and TFAM (*p* < 0.05) in the HG + Q + CC group was lower than those in the HG + Q group, whereas there was no difference in SIRT1 level (*p* > 0.05). These results indicated that quercetin activated the APMK/PGC-1α pathway in HG-exposed RSC96 cells, consistent with the results of animal experiment. Furthermore, the activation effect of quercetin on the APMK/PGC-1α pathway was similar to that of A769662, but partially blocked by AMPK inhibitor Compound C.

## Discussion

In our study, quercetin was found to have a neuroprotective effect on hyperglycemia-induced peripheral neuropathy by alleviating mitochondrial injury *in vivo* and *in vitro*. Administration of quercetin for continuous 6 weeks prominently improved the paw withdrawal threshold, nerve conduction velocity, and the pathologic changes in the peripheral nervous system of DPN rats. We also observed that quercetin alleviated the morphological damages of mitochondria in both the sciatic nerves of DPN rats and the HG-exposed Schwann cells. Furthermore, quercetin was also found to correct the decreased ATP generation under hyperglycemic conditions. Therefore, we speculated that the neuroprotective effect of quercetin might be associated with the mitochondrial energy metabolism, and the subsequent study was carried out to reveal the underlying mechanism.

**FIGURE 10:**
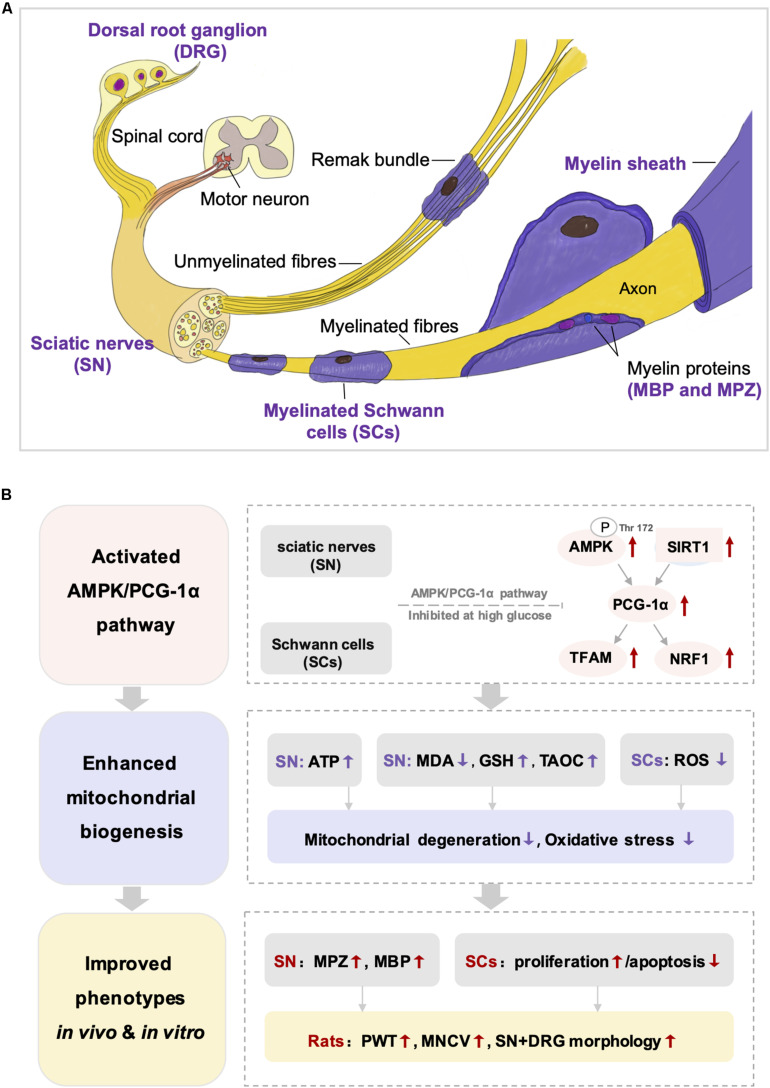
The structure of peripheral nervous system **(A)** and the integrated view of the quercetin treatment on DPN **(B)**. SN, sciatic nerves; SCs, Schwann cells; DRG, dorsal root ganglion; MDA, malondialdehyde; GSH, reduced glutathione: TAOC, total antioxidant capacity; ROS, reactive oxygen species; MPZ, myelin protein zero; MBP, myelin basic protein; PWT, paw withdrawal threshold; MNCV, motor nerve conduction velocity.

Adenosine 5′-monophosphate (AMP) activated protein kinase, a widely expressed cellular energy sensor, plays a critical role in regulating energy homeostasis (Wang et al., 2018). Under conditions of energy starvation, the increase of AMP/ATP ratio activated AMPK by stimulating phosphorylation of Thr172, which was a key residue in the α-subunit of the AMPK complex (Herzig and Shaw, 2018). Then, AMPK phosphorylated downstream targets to increase ATP production and decrease ATP consumption (Herzig and Shaw, 2018). Recent studies have confirmed that the activation of AMPK was inhibited in the peripheral nervous system of rodent models with type 1 or type 2 diabetes (Fernyhough, 2015; Yerra et al., 2018; Wang et al., 2018; Atef et al., 2019), which was in accordance with our findings that the AMPK levels (P-AMPK and AMPKα) in both SN of DPN rats and HG-exposed Schwann cells model were remarkably reduced. As shown in Figure 10, Schwann cell is the predominant cell type in the peripheral nervous system to format myelin sheath and promote regeneration after nerve injury (Goncçalves et al., 2017; Beirowski, 2019). Mitochondrial metabolism of Schwann cell is critical to meet the high metabolic demand during nerve myelination (Ino et al., 2015). In our studies, the distinct mitochondrial degeneration in both SN of DPN rats and HG-exposed Schwann cells was observed, accompanied by the decreased level of ATP. In addition, the expression of two myelin proteins, MPZ and MBP, was significantly decreased in DPN rats, indicating impaired myelin sheath and dysfunction of Schwann cell. Collectively, AMPK-mediated mitochondrial injury of Schwann cells may contribute to demyelination and axonal dystrophy, which are involved in the pathogenesis of DPN.

Some natural products, such as resveratrol (Roy Chowdhury et al., 2012), berberine (Yerra et al., 2018), and metformin (Wang et al., 2018), have been discovered as AMPK activators that alleviated the experimental DPN by improving oxidative stress, neuro-inflammation, and mitochondrial dysfunction. Quercetin, a natural flavonoid, has been found to increase the AMP/ATP ratio as an AMPK activator to promote the stimulation of glucose uptake in skeletal muscle cells (Dhanya et al., 2017). Some studies observed the therapeutic potential of quercetin against cognitive deficits and inhibit neuronal injury in DRG and the central nervous system (Shi et al., 2013; Dong et al., 2014; Pei et al., 2016; Zhang et al., 2016). In our study, we demonstrated that quercetin activated AMPK in the SN of diabetic rats and HG-exposed Schwann cells for the first time.

PGC-1α, a key regulator of mitochondrial biogenesis, could be phosphorylated by the activation of AMPK (Austin and St-Pierre, 2012). Decrease of PGC-1α in diabetes further caused mitochondrial dysfunction and in turn amplifies the toxic effects of hyperglycemia on the nervous system (Roy Chowdhury et al., 2012; Yerra et al., 2017, 2018). Consistent with previous studies, our study showed that PGC-1α expression was decreased in SN of STZ-induced diabetic rats and HG-exposed Schwann cells. The downstream transcription factors nuclear respiratory factor 1 (NRF1) and mitochondrial transcriptional factor A (TFAM) could be activated by PGC-1α and in turn promote mitochondrial respiration and replication of the mitochondrial genome (Kelly and Scarpulla, 2004; Choi et al., 2014; Chen et al., 2020). Impaired mitochondrial homeostasis in peripheral nervous system under hyperglycemic conditions were closely associated with insufficiency of NRF1 and TFAM (Yerra et al., 2017, 2018). The present research showed that quercetin elevated the PGC-1α, TFAM, and NRF1 levels, as well as improved the ATP generation and pathologic changes of mitochondria, indicating that the aberrant mitochondrial biogenesis under hyperglycemic condition was corrected by quercetin *in vivo* and *in vitro*.

Sirtuin 1 (SIRT1), a nicotinamide adenine dinucleotide (NAD)-dependent protein deacetylase, is another sensor of energetic metabolism as it senses NAD^+^/NADH ratio under the condition of nutrient consumption (Fernyhough, 2015). SIRT1 increased the activity of PGC-1α by deacetylation to enhance mitochondrial biogenesis (Zhang et al., 2017). High glucose suppressed the deacetylase activity of SIRT1 by consumption of NAD^+^ (Fernyhough, 2015; Yu et al., 2016). The subsequent acetylation of PGC-1α led to suboptimal mitochondrial function and biogenesis and inability of adapting to cellular energetic demand (Fernyhough, 2015). Significant reduction in levels of SIRT1 and PGC-1α were observed in HG-exposed neurons and SN of diabetic rats (Yerra et al., 2017, 2018). In this study, we also found a reduction on SIRT1 and PGC-1α expression in SN of DPN rats and HG-exposed RSC96, which was up-regulated by quercetin. In addition, our study found that co-treatment of quercetin and AMPK inhibitor brought no significant changes of the SIRT1 level, indicating that quercetin might active SIRT1 independent of AMPK.

The SIRT1/PGC-1α axis also modulates mitochondrial redox homeostasis. Elevated levels of PGC-1α, which can change the composition of peroxisomes and mitochondria, promoted the activity of tricarboxylic acid (TCA) cycle and the ability of anti-oxidation (Austin and St-Pierre, 2012). Up-regulation of the SIRT1/PGC-1α pathway has been reported to enhance antioxidant defenses in cardiomyocytes exposed to high glucose, while SIRT1 inhibitor led to reduced PGC-1α and elevated ROS level (Waldman et al., 2018). Previous studies have revealed that the stimulation of SIRT1/PGC-1α pathway could elevate the cellular ROS-detoxifying capacity coupled with increasing the mitochondrial ATP production (Austin and St-Pierre, 2012; Yerra et al., 2017, 2018; Waldman et al., 2018). Our study also found that quercetin treatment suppressed lipid peroxidation and enhanced the antioxidative system for the decline in MDS level and increase in TAOC and GSH. The production of ROS was also reduced by quercetin *in vitro*. These results suggested that the up-regulation of PGC-1α and SIRT1 by quercetin relieved systemic and peripheral nervous oxidative stress. Notably, the improvement of oxidative injury further inhibited DNA fragmentation of Schwann cells under high-glucose conditions (Figure 10).

Taken together, these results suggested that mitochondrial injury plays an important role in the onset and progression of DPN. The restriction of the AMPK/SIRT1/PGC-1α axis induced by hyperglycemia led to mitochondrial injury of Schwann cells and subsequently resulted in demyelination changes and axonopathy. The present study disclosed that quercetin improved the abnormal mitochondrial biogenesis and energy metabolism under diabetic conditions through activating AMPK and SIRT1, and in turn promoting PGC-1α and downstream NRF1 and TFAM (Figure 10). Meanwhile, we realized that there are some limitations in our study. We should further determine the bioenergetics profile of Schwann cells under high-glucose conditions and observe the impact of quercetin on oxygen consumption rate. Measurement of enzymatic activity of mitochondrial complexes could also be performed to provide a more comprehensive view.

## Conclusion

In this study, quercetin was found to renovate the experimental DPN *in vivo* and *in vitro*. The underlying mechanism was closely associated with the up-regulation of the AMPK/PGC-1α axis and the subsequent induction of downstream changes to restore the abnormal mitochondrial biogenesis under hyperglycemic conditions. These results highlighted the neuroprotective potential of quercetin and revealed the probable pharmacological mechanism against DPN.

## Data Availability Statement

The raw data supporting the conclusions of this article will be made available by the authors, without undue reservation.

## Ethics Statement

The animal study was reviewed and approved by the Institutional Animal Care and Use Committee of the Peking Union Medical College Hospital.

## Author Contributions

XL: conceptualization and methodology. GT: supervision. QZ: data curation, formal analysis, and writing—original draft. WS: validation and visualization. BZ, QS, XS, and BY: investigation. JX: resources. All authors: read and approved the final manuscript.

## Conflict of Interest

The authors declare that the research was conducted in the absence of any commercial or financial relationships that could be construed as a potential conflict of interest.

## Supplementary Material

Supplementary Figure 1 Effect of different high glucose concentrations for 48 h on the proliferation of RSC96 cells evaluated by CCK-8 assay. Data are shown in mean ± SEM (*N* = 5). **p* < 0.05; ***p* < 0.01; ****p* < 0.001; ns, no significance with *p* > 0.05.

Supplementary Figure 2 The bar graph exhibits the effect of quercetin (5, 10, and 20 μM) on reactive oxygen species ROS generation **(A)** and cell proliferation activity of rats Schwann cells line (RSC96) cells **(B)**. Data are expressed as mean ± SEM (*N* = 5). **p* < 0.05; ***p* < 0.01; ****p* < 0.001; ns, no significance with *p* > 0.05.

## Abbreviations

AMPK: adenosine 5′-monophosphate activated protein kinase
DM: diabetes mellitus
DPN: diabetic peripheral neuropathy
DRG: dorsal root ganglion
MDA: malondialdehyde
TFAM: mitochondrial transcription factor A
MNCV: motor nerve conduction velocity
MBP: myelin basic protein
MPZ: myelin protein zero
NRF1: nuclear respiratory factor 1
PGC-1α: peroxisome proliferator-activated receptor-γ coactivator-1α
ROS: reactive oxygen species
GSH: reduced glutathione
SN: sciatic nerve
SIRT1: Sirtuin 1
STZ: streptozotocin
TAOC: total antioxidant capacity
TEM: transmission electron microscopy
LKB1: liver kinase B1.
