# Supplementary material for: Quercetin Attenuates Diabetic Peripheral Neuropathy by Correcting Mitochondrial Abnormality via Activation of AMPK/PGC-1α Pathway in vivo and in vitro
Source: Front Neurosci. 2021 Mar 3;15:636172. doi: 10.3389/fnins.2021.636172 (PMC7966726; doi:10.3389/fnins.2021.636172)
Supplement: Supplementary Figure 1 — Effect of different high glucose concentrations for 48 h on the proliferation of RSC96 cells evaluated by CCK-8 assay. Data are shown in mean ± SEM (N = 5). *p < 0.05; **p < 0.01; ***p < 0.001; ns, no significance with p > 0.05. [file Table_1.DOCX]

**Supplementary Material**

# Quercetin Attenuates Diabetic Peripheral Neuropathy by Correcting Mitochondrial Abnormality via Activation of AMPK/PGC-1α Pathway in vivo and in vitro

**Qian Zhang^1†^, Wei Song^2^**^†^**, Bingjia Zhao^1^, Jun Xie^1^, Qing Sun^1^, Xiaohu Shi^1^, Bin Yan^1^, Guoqing Tian^1^, and Xiaochun Liang^1*^**

**^1^**Department of Traditional Chinese Medicine, Peking Union Medical College Hospital, Peking Union Medical College, Chinese Academy of Medical Sciences, Beijing, China, 100730

**^2^**Medical Research Center, Peking Union Medical College Hospital, Peking Union Medical College, Chinese Academy of Medical Sciences, Beijing, China, 100730

**^*^Correspondence:** Prof. Xiaochun Liang, Department of Traditional Chinese Medicine, Peking Union Medical College Hospital, No. 1 Shuaifuyuan Wangfujing Dongcheng District, Beijing, China, 100730 Phone 010-69155344 E-mail [Xiaochun_Liang@yeah.net](mailto:Xiaochun_Liang@yeah.net)

†These authors have contributed equally to this work.


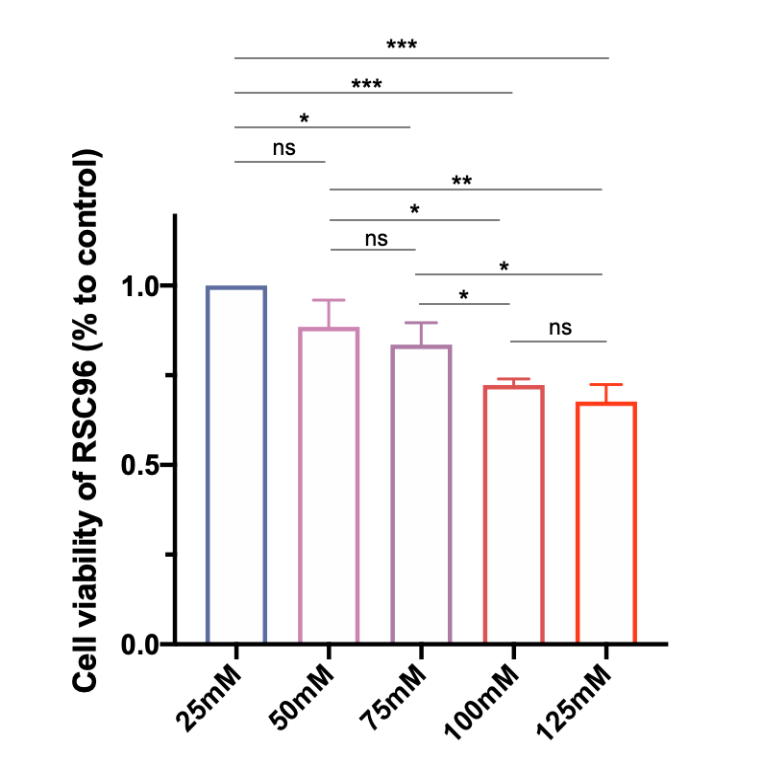


**SUPPLEMENTARY FIGURE 1 | Effect of different high glucose concentrations for 48h on the proliferation of RSC96 cells evaluated by CCK-8 assay.** Data are expressed as mean ± SEM (N = 5). **p*<0.05; ***p*<0.01; ****p*<0.001; ns: no significance with *p*>0.05.

**SUPPLEMENTARY FIGURE 2 | The bar graph exhibits the effect of quercetin (5μM, 10μM, and 20μM) on reactive oxygen species ROS generation (a) and cell proliferation activity of rats Schwann cells line (RSC96) cells (b)**. Data are expressed as mean ± SEM (N = 5). **p*<0.05; ***p*<0.01; ****p*<0.001; ns: no significance with *p*>0.05.
